# ENHANCE: The Center for Enhancing Neurocognitive Health, Abilities, Networks, & Community Engagement

**DOI:** 10.1093/geroni/igab046.732

**Published:** 2021-12-17

**Authors:** Sara Czaja, Walter Boot, Michelle Bourgeois

## Abstract

Approximately 9 % of those aged 65 and over have a cognitive impairment due to a variety of causes including Alzheimer’s disease and other forms of dementia, Mild Cognitive Impairment (MCI), Parkinson’s disease, traumatic brain injury (TBI), and stroke. Few technology solutions have been directed towards supporting older adults with cognitive impairments and the literature regarding the efficacy of these solutions is sparse. In this symposium, we describe our new Center called ENHANCE (Enhancing Neurocognitive Health, Abilities, Networks, and Community Engagement), which is focused on developing technology support for aging adults with a cognitive impairment due to MCI, TBI, and Stroke. Sara Czaja will provide an overview of the conceptual framework, goals, and structure of ENHANCE, and describe the STRUMM project, that focuses on the design and evaluation of an innovative intelligent adaptive software package aimed at providing cognitive and social support to aging adults with cognitive impairments. Wendy Rogers will discuss the ENACT project, a longitudinal needs assessment focusing on understanding the needs, challenges, and support preferences of our target population and informal caregivers. Neil Charness will describe the AUGMENT development project, which is concerned with developing an instructional support tool for mobility activities, such as wayfinding, locating, and using transportation services. Finally, Walter Boot will discuss the DREAM development project, which is focused on developing a technology-based cognitive aid to support prospective memory activities. Michelle Bourgeois will serve as the discussant for the symposium and highlight the unique opportunities and challenges associated with ENHANCE.

